# Can “the expanded free maternity services” enable Kenya to achieve universal health coverage by 2030: qualitative study on experiences of mothers and healthcare providers

**DOI:** 10.3389/frhs.2024.1325247

**Published:** 2024-09-10

**Authors:** Stephen Okumu Ombere

**Affiliations:** ^1^ Centre for the Advancement of Scholarship, University of Pretoria, Pretoria, South Africa; ^2^ Department of Sociology and Anthropology, Maseno University, Kisumu, Kenya

**Keywords:** free maternity services, Kenya, out-of-pocket expenditure, social protection, universal health coverage

## Abstract

**Introduction:**

Universal health coverage is a global agenda within the sustainable development goals. While nations are attempting to pursue this agenda, the pathways to its realization vary across countries in relation to service, quality, financial accessibility, and equity. Kenya is no exception and has embarked on an initiative, including universal coverage of maternal health services to mitigate maternal morbidity and mortality rates. The implementation of expanded free maternity services, known as the *Linda Mama* (Taking Care of the Mother) targets pregnant women, newborns, and infants by providing cost-free maternal healthcare services. However, the efficacy of the *Linda Mama* (LM) initiative remains uncertain. This article therefore explores whether *LM could enable Kenya to achieve UHC*.

**Methods:**

This descriptive qualitative study employs in-depth interviews, focus group discussions, informal conversations, and participant observation conducted in Kilifi County, Kenya, with mothers and healthcare providers.

**Results and discussion:**

The findings suggest that *Linda Mama* has resulted in increased rates of skilled care births, improved maternal healthcare outcomes, and the introduction of comprehensive maternal and child health training for healthcare professionals, thereby enhancing quality of care. Nonetheless, challenges persist, including discrepancies and shortages in human resources, supplies, and infrastructure and the politicization of healthcare both locally and globally. Despite these challenges, the expanding reach of *Linda Mama* offers promise for better maternal health. Finally, continuous sensitization efforts are essential to foster trust in Linda Mama and facilitate progress toward universal health coverage in Kenya.

## 1 Introduction

Health is a prerequisite, contributor, and indicator of sustainable development and is core to driving the 2030 Agenda (1, 2). Globally, Universal Health Coverage (UHC) has been labeled as a platform that unites health systems development across various countries (3–5). Thus, the upsurge of UHC as a global health policy agenda embedded in the sustainable development goals (SDGs) symbolizes a paradigmatic shift in global health. UHC set into motion a language of unanimity, inclusion, and social justice and championing the right of “everyone” to access and utilize the healthcare they need “without financial difficulties” (6). UHC is ultimately a progressive and ambitious goal characterized by attaining impartiality through three key scopes, namely, service coverage, population coverage, and cost-sharing (6–8). Thus, the emphasis of these broader objectives aims to offer a holistic method for tackling the unnerving health systems shortcomings faced across various settings, as stipulated within the global SDGs (9–11).

African countries have embarked on reforming health financing to avert catastrophic health expenditures to increase the utilization of maternal health services and minimize maternal deaths. For instance, Tanzania, Senegal, and Morocco removed user fees for normal birth and cesarean sections in public health facilities. Burundi and Ghana also have a free maternity policy that is extended to the private sector (12–14). Mali and Benin introduced a free cesarean section policy in all public health facilities, both public and private not-for-profit health facilities (15, 16). In Uganda and Zambia, the user fee removal policy in all public health facilities improved maternal health outcomes (17). Burkina Faso has universal care in public and selected private facilities for all expectant women (18).

As one of the central focus of the SDGs, UHC is a critical priority in Goal 3, which aims to “ensure healthy lives and promote the well-being of all at all ages” (5). According to the World Health Organization (19), achieving these goals requires the government's action in terms of budgeting to offer financial risk protection to the poorest, the healthy, and those who are ill (20, 21). In its simplest form, universal health coverage is a system in which everyone in a society can get the healthcare services they need without incurring financial hardship (22). UCH is one of Kenya's key pillars of Vision 2030 plan for socioeconomic growth. Kenya aimed to subsidize all costs for essential health services and catastrophic medical expenses for households to half by 2022 (23, 24). Moreover, reducing and eliminating pregnancy-related mortality, ending preventable newborn and child mortality, and achieving Universal Health Coverage remain crucial targets and priorities for realizing the sustainable development goals (SDGs) in Kenya. Various reforms in the health sector in Kenya have sought to achieve the above SDG targets by reducing catastrophic expenditure on maternity care and improving the quality of healthcare service delivery (25, 26). Being part of the reform linked to the UHC agenda, there were three facets targeted for improvements, namely, population, services, and direct costs, envisaging that every person would have access to the entire range of quality health services and care they needed, whenever and wherever they needed them, without financial hardship (22, 26). The *Linda Mama* policy was mainly implemented to achieve the three facets.

Since 2018, the government of Kenya has endeavored to broaden the scope of healthcare accessibility through the implementation of complementary healthcare services and the augmentation of health insurance coverage (25, 27). Although these progressive endeavors are commendable, they are superimposed upon a backdrop of healthcare histories, citizenship, and government responsibility that have been largely defined by exclusionary practices, discriminating policies, a patronage-driven political culture, and socioeconomic stratification, all of which pose significant obstacles to the achievement of universal healthcare access (6). Health is a fundamental human right enshrined in the Constitution of Kenya (2010), and to actualize this, Kenya established a Health Policy 2014–2030 whose primary objectives are “equitable, affordable, and quality health and related services at the utmost achievable standards for every Kenyan” (28).

Nevertheless, to propel the UHC agenda, the government of Kenya introduced free maternity services (FMS) in all public health facilities in June 2013 and abolished delivery fees in all public health facilities (25, 29). The expanded free maternity care program dubbed “Linda Mama” was rolled out in October 2016 by the National Government (30, 31)*.* Management of the program was handed to the National Hospital Insurance Fund (NHIF) to increase the timely processing and payment of claims (26, 32). It provides essential health services and is accessed by all in the targeted population based on need and not the ability to pay (33). *Linda Mama* aims to achieve universal access to maternal and child health services and drive Kenya toward UHC (30, 31, 34–36).

The main goals of *Linda Mama* include enhancing the availability and improving the use of skilled maternal and newborn healthcare services, reducing monetary barriers to access healthcare, and facilitating progress to UHC (29, 34, 37). This also aligns with the opinions of the African Union that supports exempting expectant women and children under the age of five from paying user fees at the point of service (38). The program aims to eliminate any fees associated with intrapartum care at public health facilities. *Linda Mama* offers a comprehensive service package that caters to the needs of pregnant women and their children. This package is provided free of charge and includes various services such as skilled delivery care, maternity care, antenatal care, postnatal care, basic obstetric emergency care, and referral to specialized obstetric emergency care to handle any complications that may arise (34).

One key dimension of equitable access is affordability, and therefore healthcare financing of maternal health is critical (26). The Kenyan health sector is financed from public, private, and donor sources accounting for 37%, 39.6%, and 23.4% of total health expenditure, respectively. Household out-of-pocket (OOP) payments account for a large proportion (26.1%) of total health expenditure (25, 39). In 2018, 7.1% of Kenyan households incurred catastrophic health expenditures, resulting in 1 million Kenyans being pushed into poverty (29, 40). Funding for county-level functions is primarily from the national government (41). Kilifi is one of the 15 counties with the highest number of maternal deaths and the highest maternal mortality ratio contributing to over 60% of the national total (42). Major health financing in Kilifi County comes through the county government, and beyond that is provided by consumers through cost-share (37).

Earlier studies in Kenya by (40, 43) revealed that the less affluent individuals contributed a considerably larger portion of their income to healthcare compared to the wealthier ones. As a result, they have a disproportionately larger portion of their household income allocated to healthcare expenses. Moreover, recent studies also indicated that maternal deaths substantially reduced despite the implementation challenges facing FMS (25, 34, 35, 44).

While Kenya's maternal and child health status has significantly improved in the last decade, the current maternal mortality ratio (MMR) of 530 deaths per 10,000 live births is significantly higher than the world average of 223 maternal deaths per 100,000 live births (45), as is the neonatal mortality rate (NMR) of 21 deaths per 1,000 live births that is higher than the world average of 18 deaths per 1,000 (46).

Although the elimination of delivery fees in public health facilities in Kenya helps alleviate financial obstacles to the use of maternal healthcare, there is still a need to address other factors and barriers that could either support or impede the successful implementation of the FMS program, which aims to enhance the achievement of UHC by 2030 in Kenya. *Linda Mama* is one of Kenya's pro-poor policies intended to benefit the poor and vulnerable; thus, the revision of the free maternity policy was intended to reduce inequities in access to maternity services and improve service access, accountability, and operational efficiency of the program (47). Additionally, recent studies in Kilifi County focused mainly on local perspectives on the implementation of FMS (25), the effects of implementing FMS (48), and health administrator's perspectives on the implementation of FMS (49). The foregoing studies did not interrogate the expanded free maternity program and whether it could help Kenya to achieve UHC a gap that this article addresses. In this article, the argument therefore is that if the investment in *Linda Mama* does not translate into achieving its primary objective, then the realization of Vision 2030 and UHC might still be a challenge. This article utilizes the perspectives of healthcare professionals and mothers utilizing *Linda Mama* in Kilifi County to explore whether *Linda Mama could enable Kenya to achieve UHC*.

## 2 Methodology

### 2.1 Study setting

The research was conducted in Kilifi County, located in Coastal Kenya. Kilifi County is considered a dry and semidry region. More than 65% of the residents in Kilifi County frequently experience a lack of water, which negatively impacts their ability to produce food and maintain food security (50). For this study, two major referral hospitals in the county were intentionally chosen. Poverty rates in Kilifi County are estimated to be 66.7%, and approximately 67% of households suffer from food insecurity. The majority of people in Kilifi County live in rural areas (42, 50). The Giriama sub-tribe, which belongs to the larger *Mijikenda* community, is the main ethnic group in the area. The Giriama people primarily not only rely on subsistence farming but also engage in wage labor in various industries such as salt mines, palm wine production, cashew nut farming, small trade, and animal husbandry. Kilifi is one of the top 15 regions in the country that has a significant impact on maternal and perinatal mortality rates (25, 51). Kilifi County exhibits one of the highest under-five mortality rates in Kenya, with 87 deaths per 1,000 live births, despite the government's initiative to offer cost-free facility-based antenatal, delivery, and postpartum services to all women (50).

### 2.2 Study design

This descriptive qualitative study was part of a larger interdisciplinary research project called *Inclusive Growth through Social Protection in Maternal Health Programs in Kenya* (SPIKE) (37). The research examined how healthcare providers and women viewed the use of the extended maternity policy and what can be learned to support the implementation of Universal Health Coverage in Kenya. This study was conducted between March 2016 and July 2017, and follow-up interviews with mothers and healthcare providers were also conducted between June 13 and July 24, 2020 (52).

### 2.3 Sample selection and data collection methods

The study utilized purposive sampling to recruit the interlocutors. To avoid recall bias, only women who were pregnant, who had given birth 6 months before the study, and aged between 18 and 45 years were purposively selected for this study at the two referral public health facilities of Kilifi County. Moreover, follow-up interviews were done with women who initially participated in the study during long-term fieldwork and those who utilized the *Linda Mama* program. Health officials were also purposefully selected as they had been in management positions for more than one year preceding the study and were concerned with maternal and child health. The health officials described their experiences offering services through *Linda Mama* and whether this could enable Kenya to achieve UHC. Initially, the author conducted a total of 40 in-depth interviews with mothers. Later, the author managed to reach 20 of them during the follow-up interviews. The mothers were interviewed one-on-one at the health facility and their homes. The author purposively selected 10 key informants for interviews. They included five health providers (matrons) in charge of maternal and child health clinic departments within the referral health facilities. Their opinion on the implementation of the *Linda Mama* program was valuable. The follow-up interviews were done during COVID-19 over the phone (52). Interviews stopped at saturation since no additional relevant categories emerged from the research participants in this study.

The study initially utilized four focus group discussions (FGDs) conducted by the author using Swahili language. Each FGD had seven mothers purposively selected from. The FGDs were audio-recorded and transcribed by the author. The emerging themes from the interview transcripts were reviewed for accuracy by the author's PhD supervisors (*not in this article*). This also ensured reliability. Moreover, the author also conducted informal conversations with community members and participant observation both at the health facility and in the community.

### 2.4 Data analysis and presentation

The author analyzed the qualitative data through two primary methods: (a) interpreting and analyzing the ethnographic data using hermeneutics and (b) conducting content analysis on the interviews (53). The transcription process was facilitated by the assistance of a computer software called F5 transcription-free. In addition to that, the author's informal conversations, which were written in notebooks, were also coded manually and incorporated into the analysis. The author had discussions with his supervisors about the identified codes to extract common themes for the complete study. Data analysis was concluded when no new themes were discovered. The findings are presented through written descriptions. The majority of the phone conversations were recorded and transcribed at a later time. Based on the overall objectives of this article, the author identified and focused on emerging themes to include in this article.

### 2.5 Ethical considerations

Taking part in the research was voluntary and participants could leave the study without facing any consequences. Written informed consent was obtained from all the interlocutors in this study. The researcher ensured that human privacy and dignity were preserved during the data collection and analysis process. All interlocutors willingly took part in the study and none of them withdrew from the study. The study was approved by the Maseno University Ethics Review Committee under the reference number MSU/DRPI/MUERC/00206/015.

## 3 Findings

There were varying perspectives of the healthcare providers and mothers as described in the findings below.

### 3.1 Improved health facility indicators

The healthcare professionals argued that, despite *Linda Mama* having challenges, it improved health facility indicators. There was an increase in facility delivery and maternal mortality decreased as most mothers could give birth in the health facility. Funds received by health facilities played a role in improving maternity and child health indicators. *Linda Mama* reimbursements helped to purchase drugs and reduce the workload in the facility by hiring extra hands. Health providers noted that *Linda Mama* enabled all mothers to give birth in the hospital regardless of their economic status. Mothers could register for *Linda Mama* either in the health facility or those that were not registered were captured during birth in the hospital.

Despite the challenges, we have improved our health facility indicators courtesy of Linda Mama. We do encourage pregnant mothers to come for delivery whether they are registered with Linda Mama or not. We have seen positive trend and I tell you maternal mortality are reduced. It is one component that pushes the UHC agenda in the right direction. (Interview with Healthcare Provider 04)

In this Country, I can tell you Linda Mama has helped increase skilled care deliveries. Though we might have a few cases of mothers giving birth at home, I know with time all mothers will give birth in hospital. I am happy to tell you we are heading to UHC. (Interview with Health Facility Administrator 02)

### 3.2 Timely disbursement vs. inadequate human resource

Expansion of FMS and change in the management of its operations enhanced transparency. The National Hospital Insurance Fund (NHIF) enhanced the timely reimbursement of funds from the national government. The healthcare providers argued that there were improvements in many healthcare sectors. Therefore, due to the transparency from NHIF and accountability by the health facilities, the healthcare providers noted that the expanded free maternity program would likely push Kenya toward the attainment of universal financing risk protection, thus realizing UHC.

We have so many changes since the expansion of free maternity services. Mothers are treated with their children up to six months after delivery. I trust the process. NHIF provides timely reimbursement to the hospitals provided we account for all the patients and services offered. Even during COVID-19, we are receiving a lot of support from the National government. One day, UHC will be attained. (Interview with Maternity Matron, Public Health Facility 02)

Linda Mama has provided avenues for health workers to get refresher courses on changes in maternal healthcare management, improving care for mothers and the child. I tell you in our maternity wards, even though we experience stock-outs of commodities, we are doing all that is possible to adhere to standard medical protocols. I trust we are on the right path towards universal health coverage. (Interview with Healthcare Provider 01)

However, other health providers had a dissenting voice. They argued that *Linda Mama* cannot lead Kenya to realize UHC unless there are multisectoral collaborations involving different actors. Such will ensure no mother is left behind. They argued that such partnerships would be effective from when a health policy is crafted to implementation.

I tell you, even with expanded free maternity and availability of free services to pregnant mothers, some are still not coming to give birth in the health facility. We still have home births and maternal deaths reported in the villages……. we need a multisectoral collaboration with all stakeholders to push the UHC agenda. Such partnerships should be from policy crafting to implementation. (Interview with Health Administrator 01)

Healthcare providers got agitated when I asked about the expanded free maternity (*Linda Mama*). It was commonly mentioned by healthcare workers that there were inadequate human resources or manpower despite the government encouraging women to give birth for free. The numbers of beds were the same, and there were stock-outs of medicine and other supplies for delivery. The healthcare workers had to improvise equipment to ensure the mothers delivered their babies safely. For instance:

If you ask about Linda Mama, I tell you I feel so bad. Nothing has changed but we hope it will change soon. We are giving the government the benefit of doubt. We still lack nurses and personnel who can operate some machines here. I can confirm to you a time we experience stock-outs but we have to improvise ways. (Interview with Healthcare Worker 03)

### 3.3 Free not free and the need for more sensitization

The healthcare providers averred that the *Linda Mama* was not free. However, the health workers were aware of how it functions and what it catered for. It was just an improved version of free maternity, although it had some additional components incorporated. However, the healthcare providers have tried their best to influence positive outcomes in the *Linda Mama* initiative. For instance, during participant observation, I noted how healthcare providers together with the community health volunteers (CHVs) give health talks during clinic visits by pregnant mothers. The interlocutors also argued that sensitization to the community by the CHVs, maternity and antenatal and child clinic (ANC) open days, and more sensitization of male partner involvement could be the best platforms for strengthening the benefits of *Linda Mama hence realization of UHC*. This could be possible if there are additional support staff and partnering with local organizations and administration.

Free maternity is not free. Anyway if at all, we engage community members continuously, then Linda Mama will be of help to many women. But you see we are understaffed. We only rely on a few CHVs who the government does not pay. But there is a need to partner with like-minded organizations and even include the local administration in this campaign. ……. today most mothers know their right in the maternity wards. They already have a positive attitude toward FMS. (KII, Matron 06, Kilifi County)

Though informally, we have been partnering with traditional midwives for referral of pregnant mothers to health facilities. We are also working closely with community health volunteers during clinic visits and also tracing pregnant mothers in the villages. To me, I feel if we have an official collaboration with more partners, then Linda Mama will propel the UHC agenda very fast in Kenya. (KII, with Matron 02)

The UHC's central agenda is to avert financial catastrophic health expenditure. However, in this study, it emerged that the promises of free maternity services (*Linda Mama*) were not real at the grassroots level. The mothers still incurred expenses while seeking care, leading to catastrophic healthcare expenses. They had to pay for laboratory services, ultrasound, and maternal health clinic registration fees and, in some cases, had to buy medicine from the local pharmacies. Mothers argued that the expanded free maternity services only catered for delivery and six weeks postpartum. They were, however, aware that free had its implications and they had to incur some costs.

We have Linda Mama cards. It is good to say the truth, we were not told that we will pay for the laboratory services, registration fees in the clinic, and buy medicine using our little money. If you come to the clinic without money, it can be messy. (Interview with a mother aged 33 years)

I had to walk back home 15 kilometres from here to go borrow money from the neighbour. The laboratory test and scanning were a must. I did not want to lose my child like last time. So, I had to pay. In Linda Mama they don’t mention anything like charges but today I know. The laboratory, medicine and registration are not free. (FGD participant, a mother aged 29 years).

### 3.4 Linda Mama is political

Although I did not ask directly about politics and healthcare, it also emerged that politics played a critical role and *Linda Mama* was politically instigated. Mothers labeled the expanded free maternity services as a political issue that the government used to get votes. Moreover, this study shows that there was political pressure to roll out *Linda Mama*. There were challenges after the rollout of the program. They included inadequate supplies or resources and inadequate human resources, and these challenges were inadequately addressed. More babies were being professionally delivered, yet supply-side constraints compromised the quality of care. Health workers explained that *Linda Mama* was a political tool for Kenya and a way to get funds from international donors. One of the health administrators argued that *Linda Mama* is a far-fetched idea to fit the global debate and agenda on health for all. However, mothers also expressed their feelings that the expanded free maternity program was political but meant to improve maternal healthcare. The association of *Linda Mama* with politics was based on their past experiences with free maternity, which was expensive and linked to the “former governments” politics’.

Linda Mama is here and we have UHC. The two cannot be separated from the global agenda of health for all. We know it is meant for our people, but politics is central. You see how the president directed for the rollout of the free maternity services. It was for gaining political mileage. This revised FMS policy is also a global politics. (Key informant interview with a health administrator, public health facility)

Who doesn’t know what politics does? All these are statements from the president. We also vote for them based on the goodies they have given us as mothers. Linda Mama is not bad but there is politics. (Informal conversation with a mother).

## 4 Discussion

In this study, I sought to explore healthcare providers’ and women's perspectives on the utilization of the expanded free maternity policy and whether the expanded free maternity policy could lead to the attainment of UHC in Kenya. Based on findings from this study, I have endeavored to identify the key issues underpinning Kenya's UHC agenda, as well as the deviations that may limit the success of UHC in Kenya. This section discusses the major themes that emerged from this study.

Findings show that the healthcare providers’ stakeholders recognized that *Linda Mama* had a positive impact on health facility indicators in Kenya. The main strength of *Linda Mama* was that it encouraged mothers to go for antenatal visits and give birth in health facilities whether they were registered for *Linda Mama* or not. Such strength contributed to improved access to health facility deliveries and care for mothers and infants. Additionally, the *Linda Mama* program introduced comprehensive maternal and child health training for healthcare professionals, and this improved the quality of services provided to the mothers. Findings also show that there were improved diagnostic and treatment practices, better patient management, and increased adherence to medical protocols. These findings are in tandem with recent studies in Kenya (25, 26, 54) that reported that the expanded free maternity program improved facility indicators. This means that the *Linda Mama* program has made significant strides in achieving UHC in Kenya. The impact of this initiative is evident in the reduction of maternal mortality rates. Access to antenatal care has increased from 56% in 2013 to 94% in 2019, while the percentage of women who deliver under skilled care has risen from 32% to 62% in the same period (34, 54, 55). Similarly, studies in Tanzania (7), Uganda (14, 17, 56), and Burundi and Senegal (57, 58) showed that there were improved facility indicators as a result of the free maternity program.

Additionally, the healthcare providers in this study and recent studies in Kenya argued that the shift in the management of FMS and the rollout of the *Linda Mama* program enhanced transparency and accountability (34, 54, 59). Moreover, Masaba and Mmusi-Phetoe (32) described the management of the *Linda Mama* program by the National Hospital Insurance Fund (NHIF) a game-changer that increased the effectiveness in the processing and payment of hospital claims, hence transparency and accountability. However, to realize transparency, ensuring compliance with the contractual agreement between providers and purchasers is essential for the provision of quality and timely services.

Despite the strengths of *Linda Mama*, findings from this study indicate that there are still gaps and challenges in implementing UHC through the *Linda Mama* program in Kenya. Such challenges included inadequate human resources, supplies, and infrastructure. Such gaps still exist despite Muinde and Prince (6) arguing that UHC is one of the most powerful concepts that public health offers to the public. Additionally, findings also show that the implementation of UHC through the *Linda Mama* program was perceived by mothers and healthcare providers as a political tool and that it was one avenue for driving the political agenda in Kilifi County. The findings also concur with recent studies that reported that reforms in Kenya's healthcare still encountered a historically uneven healthcare system shaped by forms of politics of patronage, class inequalities, and segregation and differentiation, all of which worked against universal access (6). Thus, the inconsistencies in the promises of inclusion and realities of exclusion attracted people's attention to entrenched forms of neglect, failure, and discrepancy, leading them to question rights to healthcare, state responsibility, solidarity, and growing class inequality. However, some authors (1, 60–62) argue that UHC needs to speak to local needs and thus it should include appropriate and sustainable resourcing, which includes human capital, finance, and infrastructure for its realization and sustainability. Therefore, for Linda Mama to drive Kenya toward UHC, there is a need for intersectoral collaboration that can provide a more all-inclusive, interconnected experience in the implementation of *Linda Mama* that would create a sense of ownership and push forward the realization of a sustainable UHC in Kenya.

## 5 Strengths and weaknesses

The main strength of this study is that this is one of the first anthropological studies conducted among healthcare providers and mothers that gives a primary account of the contributions of expanded free maternity services- *Linda Mama* to the pregnancy outcomes and whether *Linda* Mama could be a sure roadmap to Kenya's attaining Universal Health Coverage (UHC) in Kilifi County. The participants might have exaggerated their responses however, I countered this weakness through repeated interviews and informal conversations. While the findings may not be generalizable beyond Kilifi County because of the heterogeneity of the counties, this study identifies significant contextual factors that may be applied among other *Mijikenda* communities with a similar setup in Kilifi County. Additionally, this study can be particularly informative to policymakers as a guide to effective evidence-based interventions that can be adopted to strengthen the implementation of *Linda Mama* in Kenya.

## 6 Conclusion

This study provides a snapshot link between *Linda Mama* and UHC in Kilifi County. Healthcare providers and mothers in this study argued that *Linda Mama* led to an increase in skilled care births, improved maternal healthcare outcomes, and introduced comprehensive maternal and child health training for healthcare professionals thus improving quality of care. Despite these accomplishments, the journey toward fully achieving universal health coverage is ongoing. Challenges still exist for achieving UHC, including perceiving *Linda Mama* as a political tool and inadequate human resources, supplies, and infrastructure. However, despite the challenges, the commitment shown by the Kenyan government to provide equitable healthcare services to its citizens, particularly the most vulnerable groups, through the Linda Mama program is commendable. The *Linda Mama* program is a shining example of Kenya's dedication to achieving universal health coverage. By prioritizing maternal and child healthcare, Kenya is committed to improving health outcomes and empowering individuals and communities. As the program expands and reaches more individuals, it promises a more prosperous future for all pregnant mothers in Kenya. From the study findings, it is clear that if the challenges facing *the Linda Mama* program are addressed, it can be an appropriate path to UHC.

## Data Availability

The original contributions presented in the study are included in the article/Supplementary Material, further inquiries can be directed to the corresponding author.
